# The use of Fionet technology for external quality control of malaria rapid diagnostic tests and monitoring health workers’ performance in rural military health facilities in Tanzania

**DOI:** 10.1371/journal.pone.0208583

**Published:** 2018-12-27

**Authors:** Akili K. Kalinga, Deus S. Ishengoma, Reginald Kavishe, Lucky Temu, Christopher Mswanya, Charles Mwanziva, Erick J. Mgina, Sarah Chiduo, Lucas Mahikwano, Saidi Mgata, Lalaine Anova, George Amoo, Eyako Wurapa, Brian Vesely, Edwin Kamau, Mark Hickman, Norman Waters, Mara Kreishman-Deitrick, Robert Paris, Colin Ohrt

**Affiliations:** 1 National Institute for Medical Research, Tukuyu Centre, Tukuyu, Tanzania; 2 Kilimanjaro Christian Medical University College, Moshi, Tanzania; 3 National Institute for Medical Research, Tanga Centre, Tanga, Tanzania; 4 Henry Jackson Foundation Medical Research International, Dar es Salaam, Tanzania; 5 Tanzania Peoples Defense Forces, Dar es Salaam, Tanzania; 6 Walter Reed Army Institute of Research, MD, Washington DC, United States of America; 7 FORGYN Health Systems Consultants LLc, Washington DC, United States of America; 8 Consortium for Health Action, Phnom Penh, Cambodia; Instituto Rene Rachou, BRAZIL

## Abstract

**Introduction:**

Internal and external quality control (QC) of rapid diagnostic tests (RDTs) is important to increase reliability of RDTs currently used to diagnose malaria. However, cross-checking of used RDTs as part of quality assurance can rarely be done by off-site personnel because there is no guarantee of retaining visible test lines after manufacturers’ recommended reading time. Therefore, this study examined the potential of using Fionet™ technology for remote RDT quality monitoring at seven clinics, identifying reasons for making RDT processing and interpretation errors, and taking corrective actions for improvement of diagnosis and consequently improved management of febrile patients

**Methods:**

The study was conducted at seven military health facilities in Mainland Tanzania and utilized RDTs capable of detecting *Plasmodium falciparum* specific Histidine-rich protein 2 (Pf-HRP2) and the genus specific Plasmodium lactate dehydrogenase (pLDH) for other species of plasmodium (*P*. *vivax*, *P*. *malariae* or *P*. *ovale*; pan-pLDH). Patients’ data and images of processed RDTs from seven clinics were uploaded on a Fionet web portal and reviewed regularly to monitor preparation procedures and visual interpretation of test results compared to automated analysis using the Deki reader of RDT. Problems detected were rapidly communicated to remote laboratory personnel at the clinic for corrective action and follow-up of patients who were falsely diagnosed as negative and missed treatment. Factors contributing to making errors in visual interpretation of RDT results were analyzed during visits to the health facilities.

**Results:**

A total of 1,367 (1.6%) out of 83,294 RDT test images uploaded to the Fionet portal had discordant test results of which 822 (60.1%) and 545 (39.9%) were falsely reported as negative and positive, respectively. False negative and false positive test results were common for a single test line in 515 (62.7%) and 741 (54.2%) tests, respectively. Out of 1,367 RDT images assessed, 98 (7.2%) had quality problems related to preparation procedures of which 95(96.9%) errors were due to putting too much blood on the sample well or insufficient buffer in the respective wells. The reasons for discrepant results included, false reporting of none existent lines in 526 (38.5%) tests, missing a faint positive line in 493 (36.1%), missing a strong positive line in 248(18.1%) and errors caused by poorly processed RDTs in 96 (7.2%) tests. Among the false negative tests (n = 822), 669 (48.9%) patients were eligible for follow–up and only 339 (48.5%) were reached and 291 (85.8%) received appropriate anti-malaria therapy.

**Conclusion:**

Fionet technology enabled remote monitoring of RDT quality issues, identifying reasons contributing to laboratory personnel making errors and provided a rapid method to implement corrective actions at remote sites to improve malaria diagnosis and consequently improved health care management of febrile patients infected with malaria.

## Introduction

Malaria rapid diagnostic tests (RDTs) have become a reliable technique for confirmation of malaria infection and providing results for guiding patient treatment [1–3].The technique is now widely used [4]especially in settings with limited resources and where microscopy is not feasible due to its well documented limitations [5–7]. RDT tests do not require sophisticated infrastructure, are easily performed and the results correctly interpreted appropriately by trained health care workers even those with limited laboratory training or experience [8].

Despite their wide use in malaria diagnosis, RDTs face multiple potential quality problems starting at manufacturing through production transportation, storage and end-user’s performance [9]. False RDT results due to problems at any of the above stages may lead to increased malaria transmission[10], unnecessary exposure to anti-malaria therapy, increased risk of a lack of treatment for other diseases that cause fevers [11,12], and development of resistance by *Plasmodium* species.The Global Malaria program of the World Health Organization (WHO) has set up a comprehensive quality control (QC) strategy at levels of production, transport and product control in national reference laboratories as well as a lot testing program [13]. Many malaria programs, however, lack quality control (QC) processes to assess the use of RDTs under field conditions in order to ensure that they are still functioning properly after exposure to variable transport and storage conditions [14–16].

According to the WHO guidelines, the QC of RDTs describes all the activities to be undertaken by a laboratory to monitor each stage of a test procedure to ensure that RDTs are performed correctly and accurately interpreted to give precise results [17]. [18]. The recently developed positive control wells containing recombinant malaria antigen is a step forward for QC of RDT kits at the point of care which will help to improve the confidence of health workers in RDT results [16,19,20]. Findings from pilot studies in field conditions in Kenya and Tanzania suggest that this means of RDT quality assurance could improve health worker adherence to RDT testing procedures [20]. The dried tube specimen (DTS) method has also been tested and can be used under field conditions where appropriate [21].

In a previous study organized a short messaging systems (SMS) based external quality assurance procedure transmitting high resolution photographs of used RDT to enhance correct reading and interpretation of RDTs among laboratory health workers [22,23]. In order to enhance improvement of RDT quality control, Fio Corporation based in Toronto, Canada, innovated the use of its Fionet platform for remote monitoring and provision of real-time feedback on RDT testing conducted by users at clinics [24]. The Fionet system uses a device called Deki Reader™ which combines standard mobile devices with custom software to gather demographic patient data, provide guidance to health care workers on conducting testing, taking pictures of completed RDT assays, transmits data over commercially available cell phone services. It also contains a web portal for uploading processed RDT images, transmission of patient demographic information, and remote storage and access of the data [25–29]. This mobile health technology platform has been successfully used in small programs for quality assurance and quality improvement of malaria diagnosis by community health workers in Kenya [24].

In this study, an assessment was conducted on the potential application of Fionet technology for remote RDT quality monitoring at seven military clinics, identifying reasons for making RDT processing and interpretation errors, and taking corrective actions for improvement of diagnosis and consequently improved management of febrile patients.

## Materials and methods

### Study area

**Fig 1:**
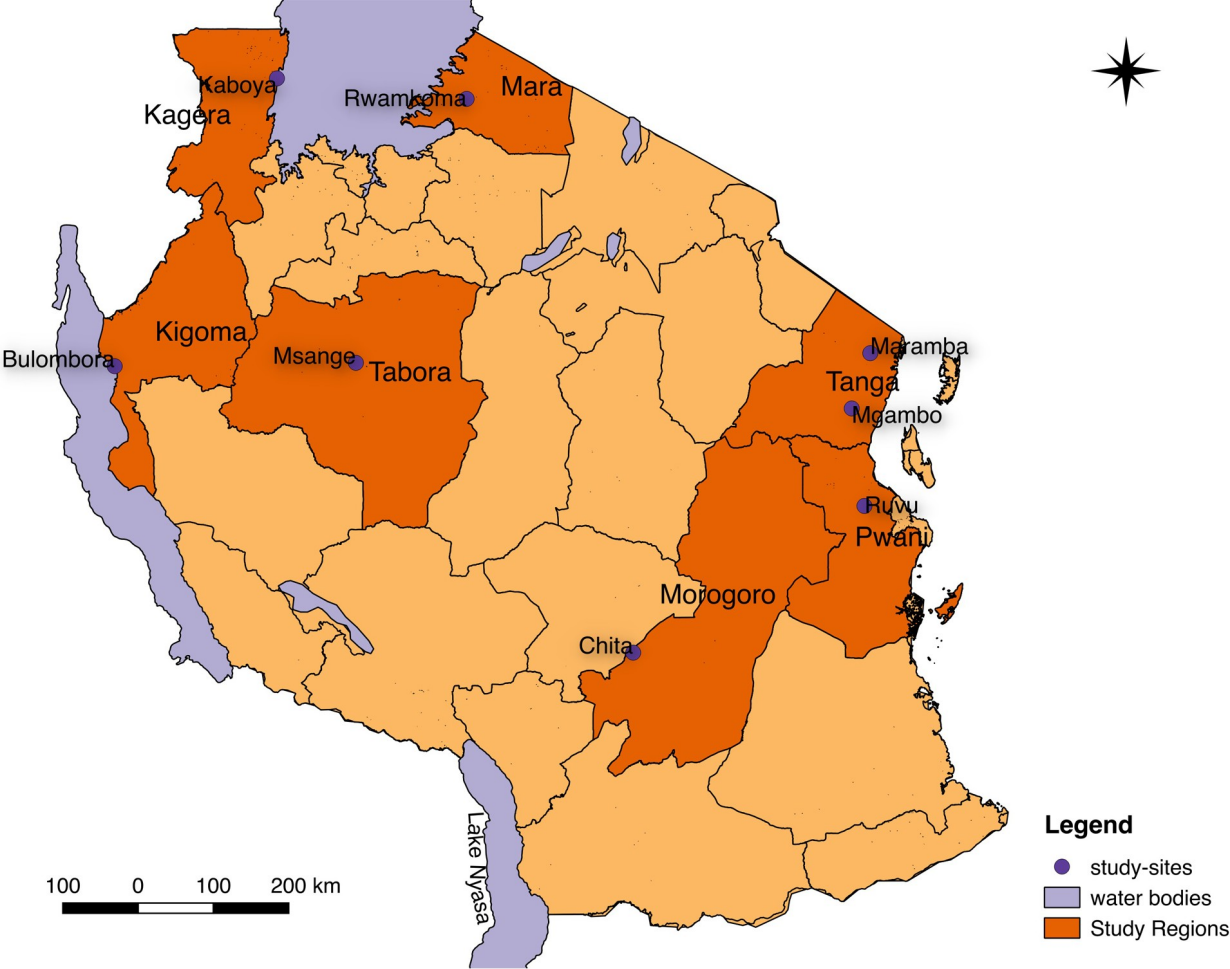
Map of Tanzania showing distribution of study sites. The map was generated using shape files and data using qGIS {version 3.2 (Bonn)} which is open source program [33].

The study was conducted in seven rural military health facilities in Tanzania (Fig 1) including Ruvu (Coastal region), Mgambo and Maramba (Tanga region), Bulombora (Kigoma region), Chita (Morogoro region), Rwamkoma (Mara region) and Msange (Tabora region). The regions have varying malaria transmission intensity as previously shown by the nation-wide surveys [30–32]. The selected health facilities are owned by the Tanzanian national service commonly known as “Jeshi la Kujenga Taifa” (JKT), which is under the Tanzania Peoples Defense Forces (TPDF), and these facilities are primarily dispensaries serving both outpatients and inpatients.

### Study design, populations and period

The study employed an analytical cross-sectional design and was nested on an ongoing larger study on the surveillance and quality improvement of malaria diagnosis in military health facilities. The study population was routine outpatients (military staff, recruits and civilians) of all ages and sex and ages seen at outpatient departments (OPDs) at the seven health facilities. This study was conducted for three years from January, 2014 to December, 2016.

### Selection of study participants

Patients who presented at an OPD with fever occurring within a 24-hour period prior to presentation were conveniently sampled for the study. Sampling was done after obtaining a voluntary written consent from patients or parents/care-takers in case of children.

### Training users on Fionet technology

A total of 16 project staff identified for data collection were trained on Fionet technology for three days. Of these, 14 were routine laboratory assistants identified as users of Deki Readers at clinic facilities and two were study investigators who worked as study monitors at the project base located in Dar es Salaam. Two laboratory assistants were selected from each of the seven study health facilities. On the first day of training, all project staff were trained on theoretical overview of Fionet technology. On the second day, laboratory assistants were trained on operating and using the Deki Readers while monitors were trained on how to remotely monitor laboratory personnel at clinic sites using the Fionet portal and mobile communication. On the third day, practical sessions were done as trials for preparing and reading RDTs, uploading data, and accessing data and reports in the portal. The study utilized a one step 05fk60 SD Bioline Malaria Antigen Pf/pan RDT cassettes from Standard Diagnostics Inc (Republic of Korea) capable of detection of malaria infection in whole blood of *Plasmodium falciparum* specific Histidine-rich protein 2 (Pf-HRP2) and the Plasmodium lactate dehydrogenase (pLDH) for other Plasmodium species (*P*. *vivax*, *P*. *malariae* or *P*. *ovale*, pan-pLDH). Monitors and laboratory personnel were also trained on how to communicate through two ways messaging between the Deki Reader and the portal as well as telephone conversations and SMS when errors were encountered and corrective actions were required. The data collection methods were piloted for one week as “extended training” and this training has been reported elsewhere [29]. Occasionally, in some health facilities, trained laboratory personnel were either transferred to other facilities or recruited for further studies and they were replaced by untrained personnel. In this scenario, untrained personnel received initial training from their peer laboratory personnel on how to use Deki Reader and perform RDTs but did not perform the tests for patients until after they were formally trained by study monitors during quarterly supportive supervision visits

### Data collection procedures

**Fig 2:**
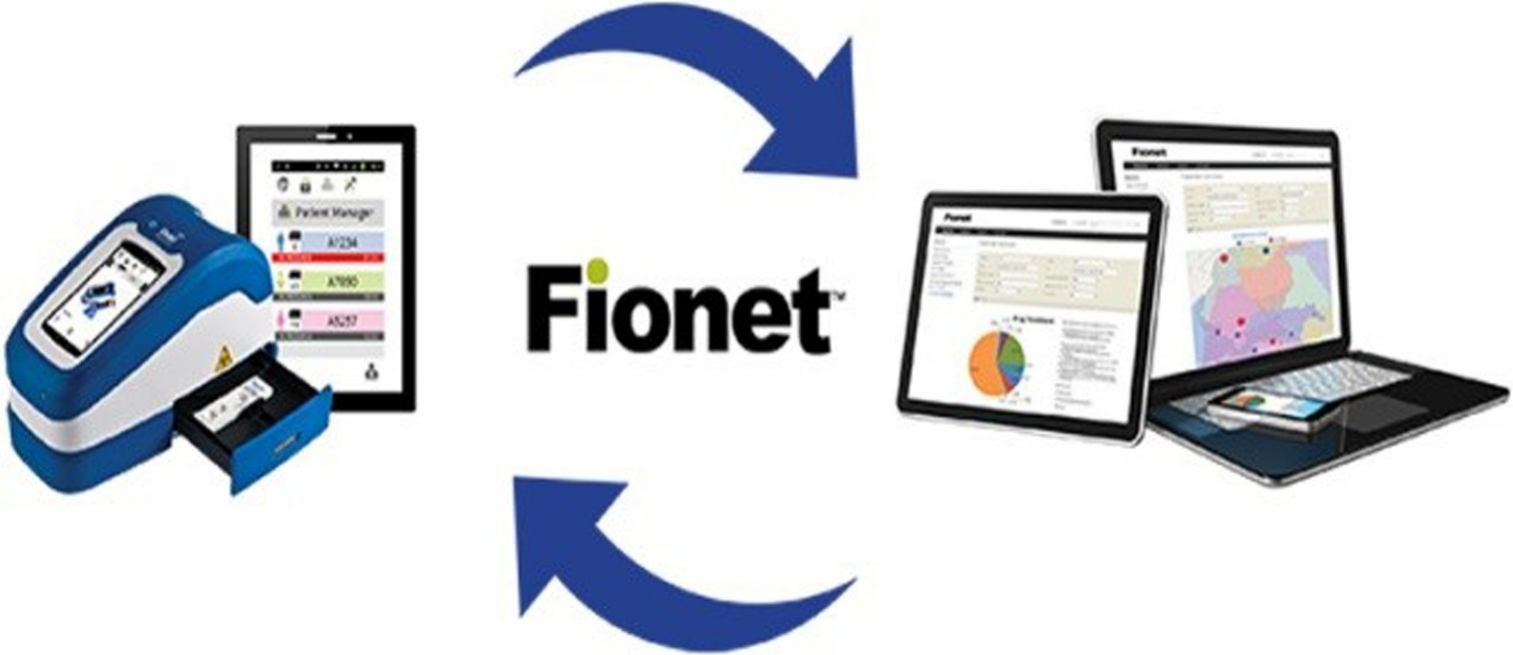
Fionet platform for data capture and quality improvement of RDT. (Reprinted from Fio Corporation marketing materials under a CC BY license, with permission from Fio Corporation, original copyright 2015).

Data collection was done using Fionet technology (Fig 2). The laboratory personnel performed RDT testing using the Deki Reader, read the results, and directly submitted patient demographic data, manual laboratory evaluation of the RDT result, automated Deki Reader evaluation of the RDT result, and high resolution RDT test images to the portal as previously described [26,28,29]

Monitors used secure Fionet accounts to review records and RDT images uploaded to the web portal. HHuman errors in preparation procedures and interpretation of test results were investigated; but errors due to manufacturing, transportation, storage and effect of antigen/antibodies on the cassettes and other testing issues were not studied. The Deki Reader was considered superior to human interpretation of RDTs and hence the difference of test results between the two methods was regarded as “discordant results”. The study monitors rapidly contacted laboratory personnel through telephone calls or alert messages, for corrective actions of any detected errors. The study monitors also communicated test results reported falsely as negative to re-check patients’ results in the laboratory register. Laboratory personnel also followed- up with patients who missed treatment to present to clinic for appropriate treatment. Patients treated on follow-up or otherwise were analyzed and confirmed during physical quarterly site visits by the study monitors. Due to limited time and other logistical problems, civilians with false negative test results did not receive a follow-up visit. After confirmation of discrepant laboratory results, some patients who missed treatment on their first clinic visit due to false negative results were treated after their second clinic visit.

Reasons contributing to errors in visual interpretation of RDT results by laboratory staff were analyzed regularly by the study monitors through reviewing images of RDTs in the portal, recorded results in the laboratory registers, review of automated analysis results, and re-interpretation of stored old RDTs (if readable) during physical visits to the study sites. The overall results were compiled to help take corrective measures to improve malaria diagnosis. During physical visits at the clinic sites, monitors also verified laboratory records and patient records to confirm patients re-tested and treated after follow-up.

### Ethical issues

The study received ethical approval from the National Medical Research Coordinating Committee (MRCC) of the National Institute for Medical Research (NIMR) and from Kilimanjaro Christian Medical University College’s (KCMUCo) Ethics Review Committee. Permission to conduct the study in military health facilities was granted by the Chief of Medical Services at the headquarters of the TPDF and the Commanding Officers (COs) of the respective military camps where the health facility was housed. Participants were informed about the nature and aims of the study, and its benefits and risks. They were also sensitized about the study and malaria diagnosis using RDT with Deki Readers before voluntarily consenting to participate. Military officers were not involved in the consenting process involving junior military personnel to avoid coercion. Only unique identification numbers for each patient and not personal identifiers such as names and initials were used in the data collection process. The national malaria treatment guidelines were used to treat patients with positive results by RDT as interpreted visually on their first clinic visit and after follow-up in case false negative results were detected by Deki Readers.

### Data management

Data were entered by using the screen interface of the Deki Reader, which was connected to the national mobile network and data encryption was used to transmit data to the secured portal. In the portal, secured data were accessed by accredited project staff (study monitors) through a password-protected website with access controlled by the study managers. The test results were transferred to Microsoft Excel and assessed to detect discrepancies between visual and DR-interpreted RDT results. These were merged with another Excel dataset that included review information from the laboratory register, results of re-reading old RDTs, reasons for false results and patient follow-up data.

The data was then checked and transferred to STATA software (STATA Corp, College Station, TX) for analysis (S1). Descriptive statistics was used to describe the basic features of the data, proportions of discordant test, RDT quality problem due to interpretation procedures. Reasons for making errors in interpretation of RDT results and their corresponding 95% confidence intervals were analyzed using the Wilson score method. Simple graphic to describe errors in RDT preparation and interpretation of RDTs by facility and years are presented. Statistical significance was reported at *p* < 0.05

## Results

### Baseline characteristics of the study population

Out of 83,294 RDT images uploaded to the web portal, 2,008 were found to have discordant results after comparing laboratory personnel derived manual RDT results against results obtained by automated analysis using Deki Readers. Through analysis by study monitors and communication with laboratory personnel at clinic sites it was revealed that laboratory personnel erroneously transposed the results of 641(0.8%) tests when entering the results in the Deki Readers. They reported results as false negative as opposed to true positive results entered into the laboratory records at the clinic sites, and these results were therefore excluded from the analysis. Thus, a total of 1,367(1.6%) out of 83,294 RDT images uploaded to the Fionet portal were confirmed to have discordant results in RDT interpretation between laboratory personnel and automated analysis by the Deki Readers. The corresponding discordant results by year were 361 (0.8%) among 46,612 in 2014, 582(2.6%) among 22,401 in 2015 and 424(2.9%) among 14,282 uploaded images in 2016.

Table 1 summarizes the characteristics of discordant results. Maramba constituted 402(29.4%) discrepant results (the highest among all study sites) while the Rwamkoma had only 72(5.3%), the lowest among all sites. Participants aged 18-25years old constituted 972(71.1%) discrepant results; the highest of all age groups. In the first year (2014), Msange and Chita sites were not included in the study while Rwamkoma had missing data in 2015. The proportions of discrepant results among patients of different age groups, sex and those from the different study facilities were significantly different (p<0.001). A total of 699(51.1%) patients with discrepant results were eligible for follow-up.

**Table 1:** Baseline characteristics of study participants with discordant RDT results.

Variables	2014 (n = 361)	2015 (n = 582)	2016 (n = 424)	Total (N = 1367)
	n(%)	n(%)	n(%)	n(%)
Sex*				
Male	266(73.7)	431(74.1)	298(70.3)	995(72.8)
Female	95(26.3)	151(25.90	126(29.7)	372(27.2)
Patients' age groups(years)*				
<5	29(8.0)	50(8.6)	34(8.0)	113(8.3)
5–17	26(7.2)	40(6.9)	25(6.0)	91(6.7)
18–25	253(70.1)	405(69.6)	314(74.1)	972(71.1)
>25+	53(14.7)	87(14.9)	51(12.0)	191(14.0)
Health facilities*				
Bulombora	104(28.8)	17(2.9)	9(1.1)	130(9.5)
Chita	0(0.0)	80(13.7)	64(15.1)	144(10.5)
Maramba	78(21.6)	228(39.2)	96(22.6)	402(29.4)
Mgambo	60(16.76)	116(19.9)	144(33.9)	320(23.4)
Msange	0(0.0)	35(6.0)	52(12.3)	87(6.4)
Ruvu	71(19.7)	106(18.2)	34(8.0)	211(15.4)
Rwamkoma	48(13.3)	0(0.0)	25(6.0)	73(5.3)
Type of discordant results*				
False negative results	245(67.9)	346(59.5)	231(54.5)	822(60.1)
False positive results	116(32.1)	236(40.5)	193(45.5)	545(39.9)
Patients eligible for follow-up**				
Eligible	200(55.4)	282(48.4)	217(51.2)	699(51.1)
Not eligible	161(44.6)	300(51.6)	207(48.8)	668(48.9)

* p≤0.001

** p≥0.001

### Evaluation of discrepancies in interpretation of RDT results by laboratory personnel compared to automated analysis

Out of the 1,367 individuals with discordant RDTs results as assessed by comparing interpretation by laboratory personnel and automated analysis by Deki Readers, a significantly higher proportion (60.1%) had false negative compared to those with false positive results (39.9%) (p<0.001). A total of 741(54.2%) were due to personnel errors associated with an inability to discern a single test line displaying positive or negative results, 483(35.3%) were associated with inability to discern either of the two positive or negative lines and 143 (10.5%) were associated with inability to discern both of the two positive or negative lines.

**Fig 3:**
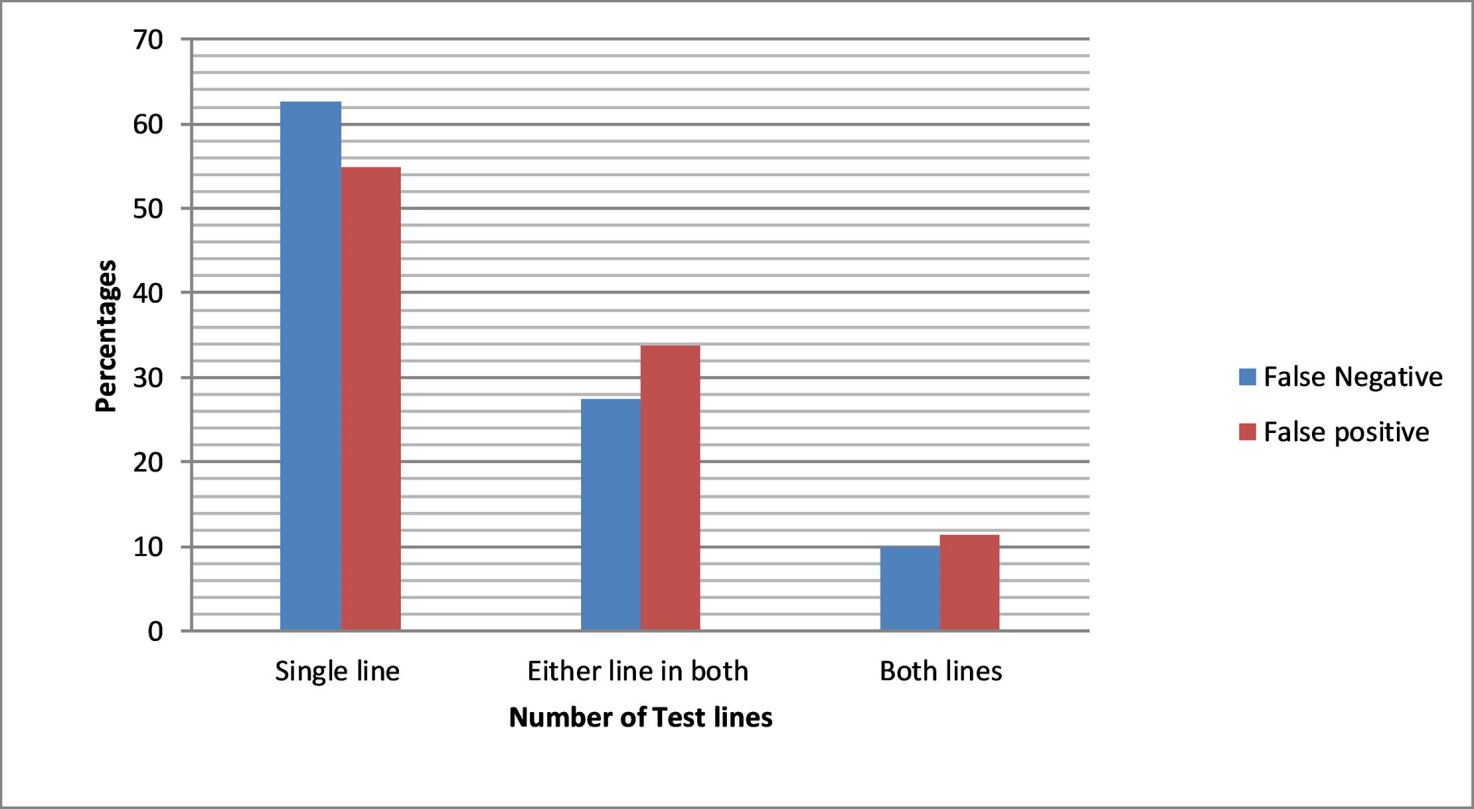
Characterization of interpretation errors of RDT by test line results.

Of the false negative results, 515(62.7%) were due to personnel errors associated with inability to discern a single test line displaying positive results, 226(27.5%) were associated with in an inability to discern either of two positive lines and 81(9.9%) were associated with an inability to discern RDTs with two positive lines (Fig 3). Of the false positive results, 299(54.9%) tests were associated with personnel falsely reporting the presence of a single test line as positive, 184(27.5%) errors were associated with falsely reporting the presence of either line of the two test lines and 62(11.4%) errors were associated with falsely reporting both test lines as being positive (Fig 3). There was a significant difference (p<0.001) in making interpretation errors for a single RDT test line compared to errors associated with determining the validity of either line of an assumed double line and errors associated with cases where both test lines were implicated.

**Fig 4:**
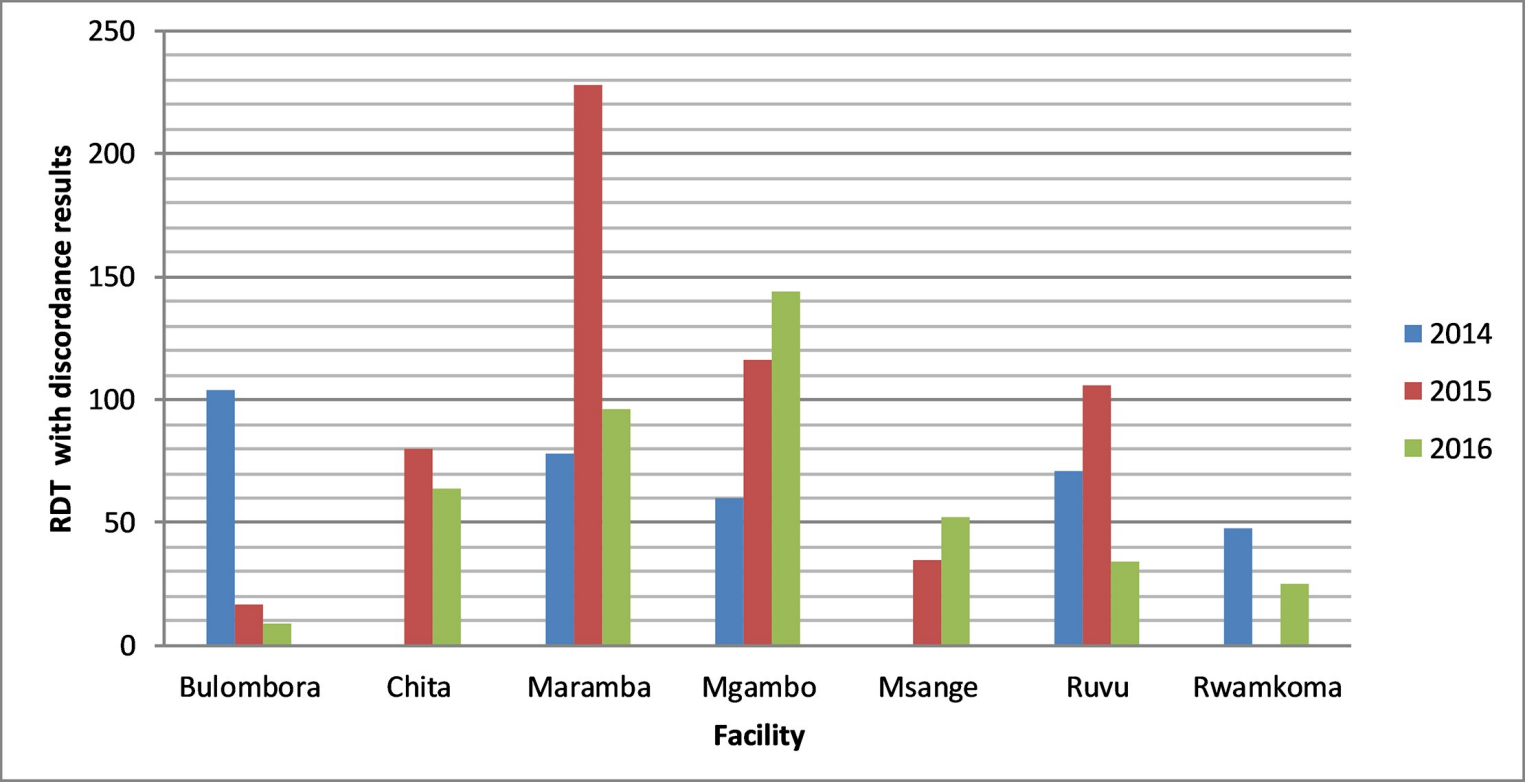
Number of RDT images with errors in interpretation reported by health facilities from 2014 to 2016.

Despite remote monitoring of the sites, RDTs with discrepant results increased from 361 (26.4%) in 2014 to 582 (42.6%) in 2015 and decreased slightly to 424 (31.0%) in 2016. Generally, an increasing trend in making interpretation errors was observed in the three years period (Fig 4)

### Reported errors in preparation procedures of RDTs

Of all the discordant results, 98(7.2%) involved review of RDT images which showed quality problems related to preparation procedures. Of these, 95(96.9%) errors were associated with errors caused by putting too much blood on the sample well or insufficient buffer in the respective wells while only 5(5.1%) were due to putting too little blood on the sample well or applying blood on walls of the wells. Out of 1367 RDT images with discordant results, 98(7.2%) had errors in preparation of which, 78(5.7%) were prepared in 2014, 18(1.3%) in 2015 and 2(0.1%) in 2016. Three out of seven study health facilities (Bulombora, Rwamkoma and Ruvu) contributed over 50% of all tests with processing errors.

**Fig 5:**
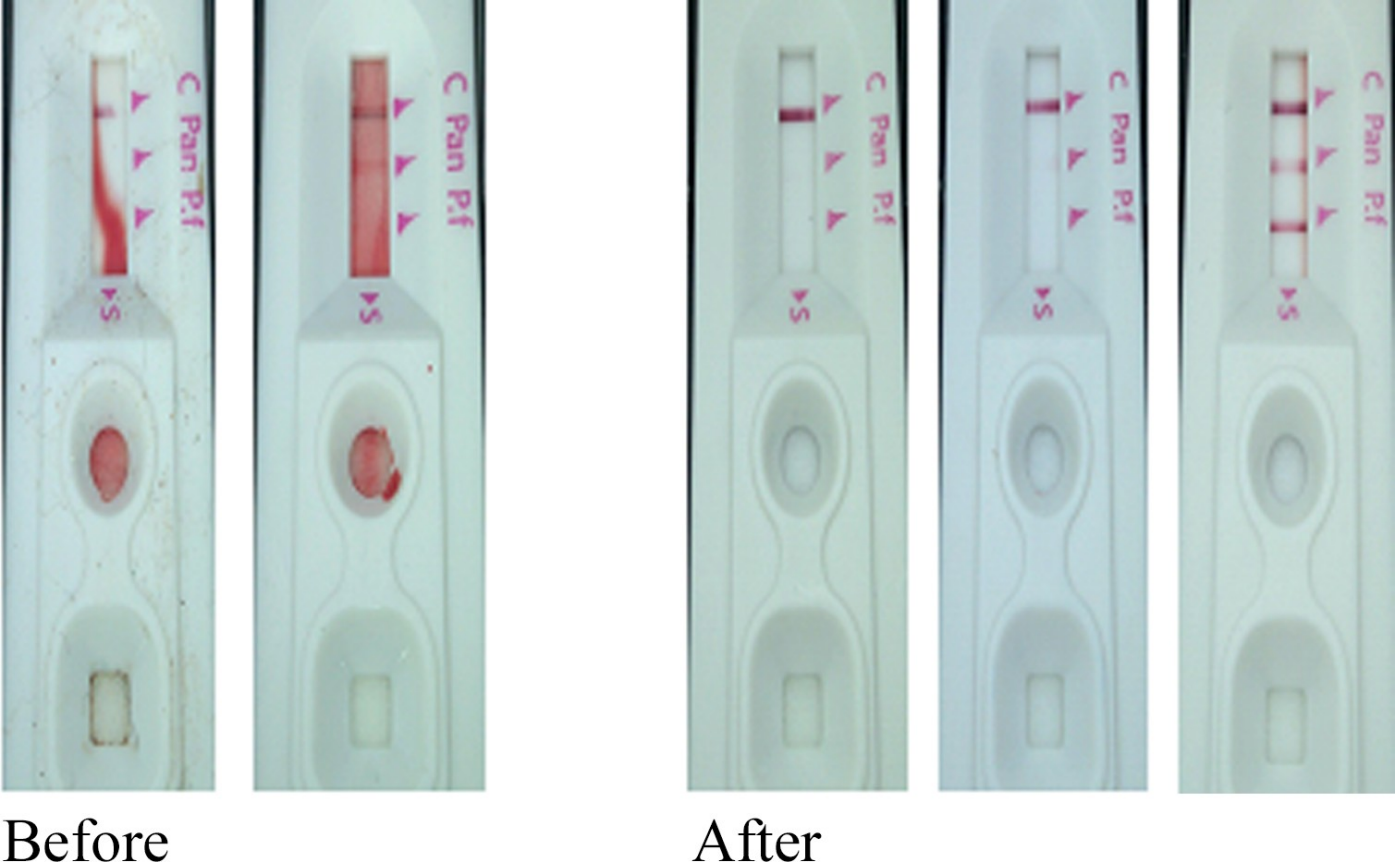
A sample set showing quality of RDTs prepared before and after remote monitoring.

**Fig 6:**
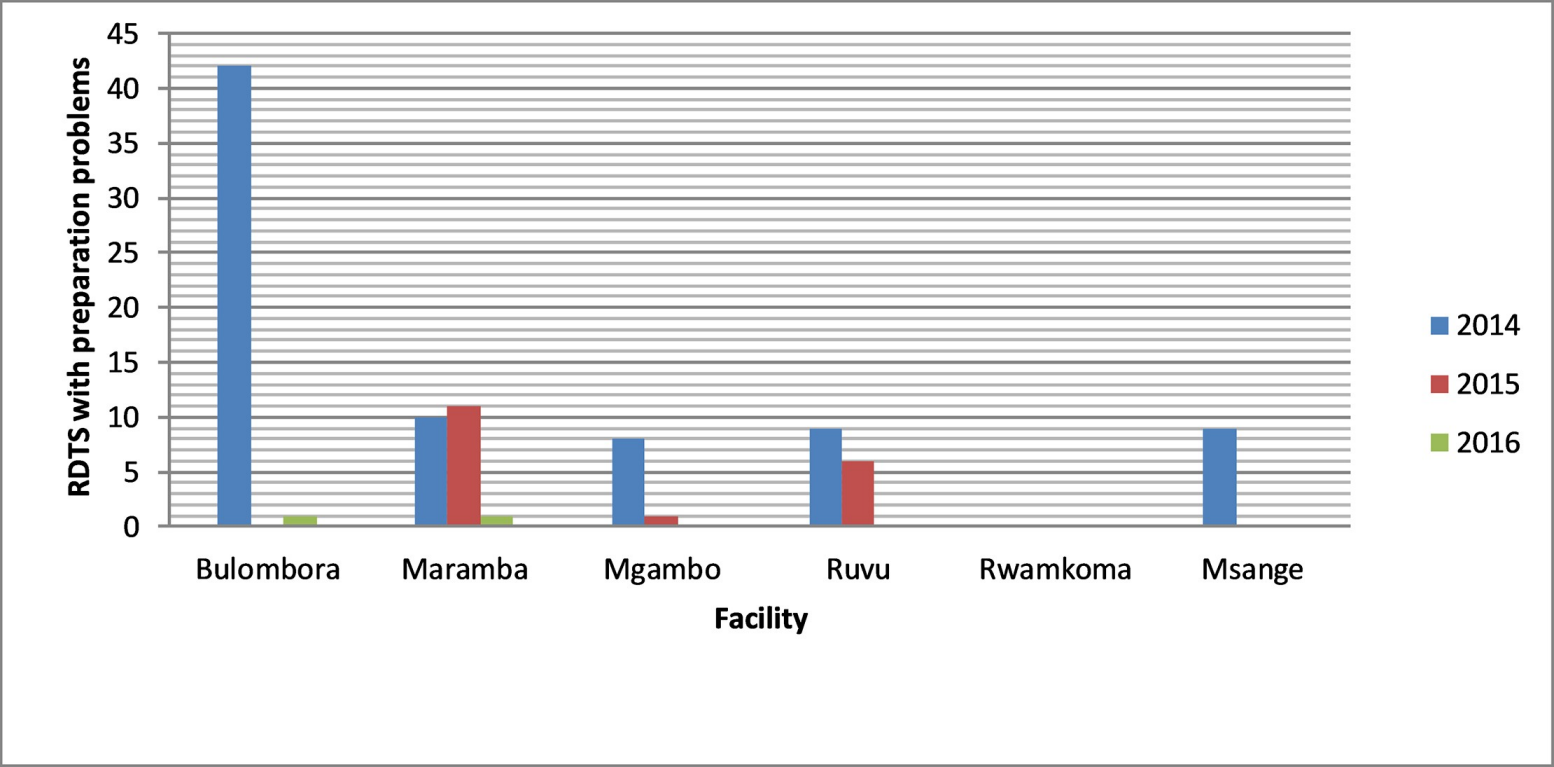
Number of RDT images with preparation errors reported by health facilities from 2014 to 2016.

In the course of remote oversight, study monitors contacted laboratory personnel for corrective actions when mistakes were detected, leading to a significant decline in preparation errors (*p* < 0.001) from 5.7% in 2014 to 1.3% in 2015 and further to 0.1% in 2016. Except for Maramba where errors in preparation of RDTs were almost steady from 2014 to 2015, there was a noticeable decrease in errors for all study sites (Figs 5 and 6). After three years of remote monitoring with Fionet technology, errors associated with preparation of RDTs were reduced by 97%.

### Reasons for making errors in interpretations of RDT results

**Fig 7:**
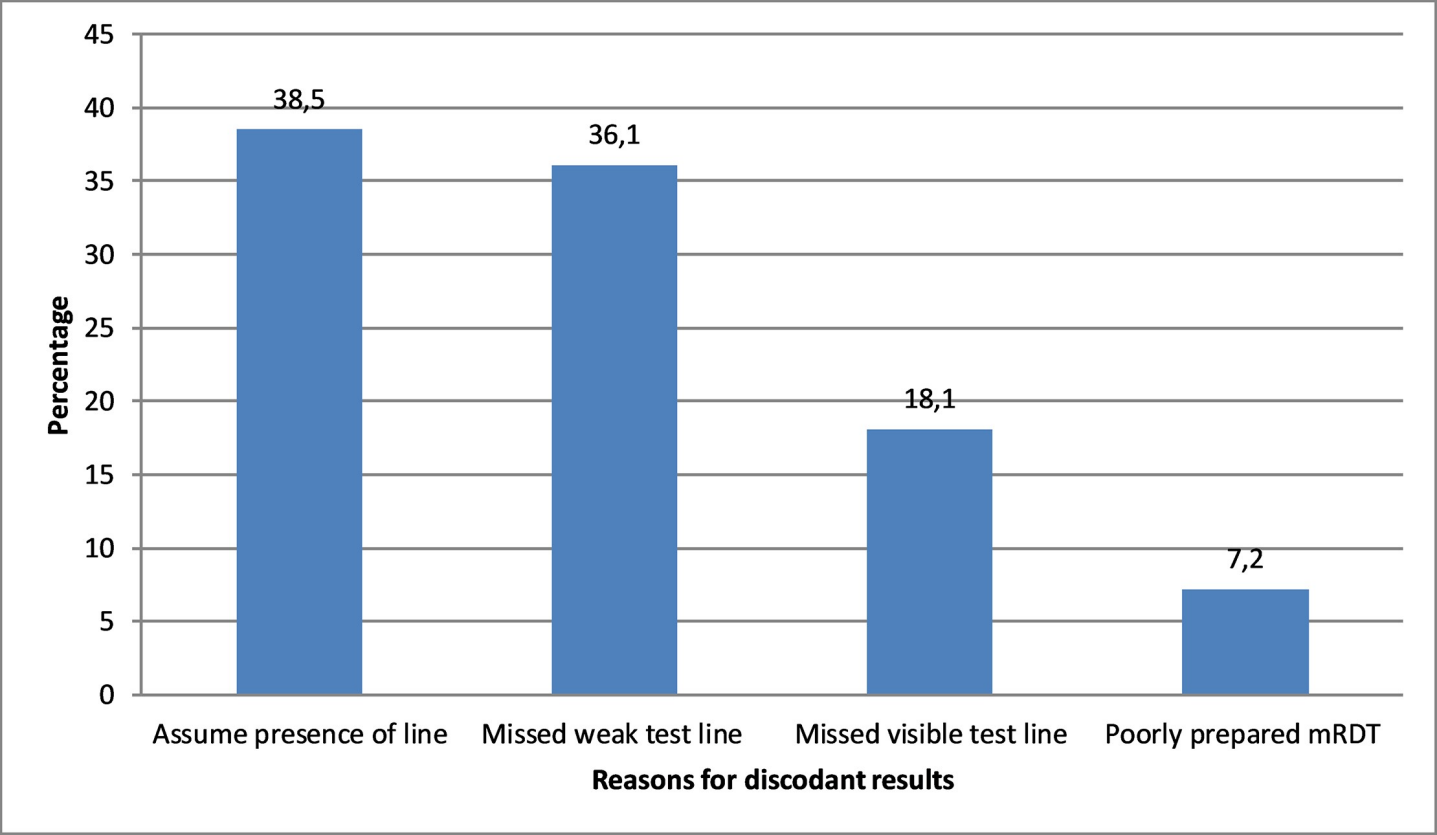
Reasons for laboratory personnel making errors in interpretation of RDT.

**Fig 8:**
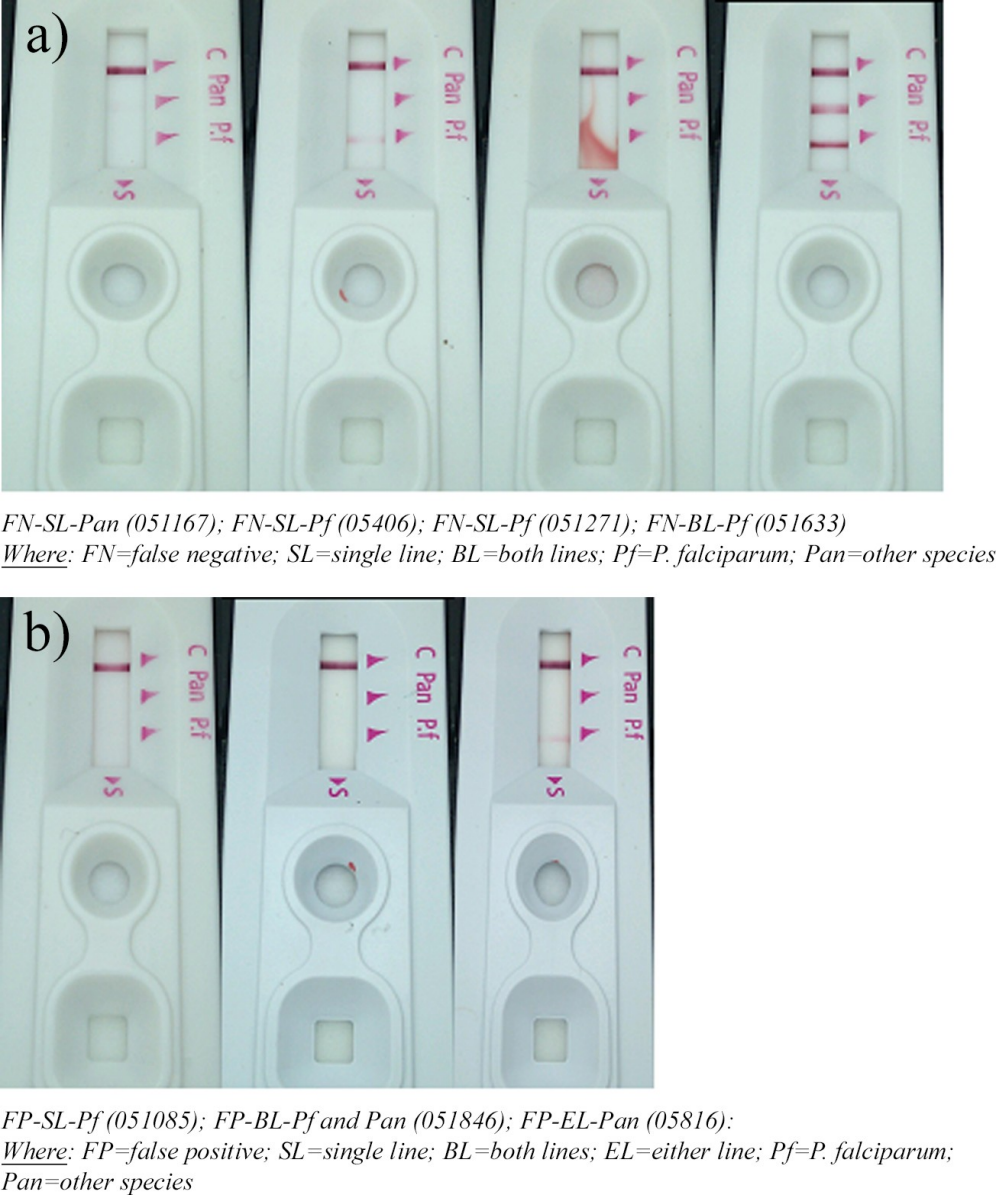
a) Examples of negative falsely interpreted test lines of RDTs at Maramba clinic in April, 2014. b) Examples of positive falsely interpreted test lines of RDTs at Maramba clinic in May, 2014.

Errors in RDT interpretations were contributed mostly (>95%) by failure to adhere to testing procedures associated with RDT preparation and interpretation of RDT results by laboratory personnel. A total of 526 (38.5%) were due to errors by laboratory personnel who falsely observed positive test line on RDTs due to over interpretation when in fact these lines were not present while in 493 (36.1%) cases, a faint positive RDT test line(s) was missed by the end user. It was also observed that 248(18.1%) errors were due to laboratory personnel failing to observe and record strong positive test lines on RDTs, and 96 (7.2%) were associated with errors due to poorly processed RDT tests. Reasons for Laboratory personnel making errors in interpretation of RDT results are shown below in the bar chart (Fig 7); Examples of image of RDTs are also shown below as negative (Fig 8A) and positive (Fig 8B) falsely interpreted respectively.

### Follow-up of patients who missed treatment on initial RDT false results

Out of 1,367 patients with discrepant test results, 699(51.1%) were eligible for follow-up to initiate malaria treatment because they were detected to be false negative RDT results after the initial tests. A total of 668(48.9%) patients were not eligible for follow-up because they had either false positive or false negative results (in one of the two lines but were reported as positive lines) and they were actually treated based on the initial RDT results. Among eligible patients for follow-up, 339(48.5%) were followed-up while 360(51.5%) were not followed-up because they were civilians coming from neighboring villages where follow-up was not logistically feasible. Of the patients who were followed-up, 291(85.8%) were treated with appropriate anti-malaria therapy while 48(14.2%) patients were not treated because of delays in communication with health workers at the clinic (Table 2). During follow-up, most of the patients were followed-up and treated on the same day the initial RDT results were reported (42.8%) or after one day (45.4%) (Table 2). There was no significant difference among health facilities and day of treatment after follow-up. However, the number of patients treated in 2015 were significantly higher (*p*< 0.001) compared to those treated in 2014 and 2016 (Table 2).

**Table 2:** Number of patients followed-up and the day of follow-up and treatment.

Variable	Same day	1day	2 days	3 days	> 3 days	Treated	Missed	Followed
	# (%)	# (%)	# (%)	# (%)	# (%)	# (%)	# (%)	#
H. facilities								
Rwamkoma	13(68.4)	5(26.3)	1(5.3)	0 (0.0)	0 (0.0)	19(95.0)	1(0.5)	20
Bulombora	17 (44.7)	14 (36.8)	4(10.5)	1 (2.6)	2 (5.3)	38(84.4)	7(15.6)	45
Maramba	32 (40.0)	41 (51.1)	6 (7.5)	1 (1.1)	0 (0.0)	80(86.0)	13(14.0)	93
Mgambo	25 (37.3)	31 (46.3)	5 (7.1)	4 (7.5)	2 (3.0)	67(95.7)	3(4.3)	70
Msange	10(33.3)	16 (53.3)	4 (13.3)	0 (0.0)	0 (0.0)	30(90.9)	3(9.1)	33
Ruvu	26(45.6)	25(43.9)	6(10.5)	0 (0.0)	0 (0.0)	57(73.1)	21(26.9)	78
All	123 (42.3)	132 (45.4)	26(8.9)	6 (2.1)	2 (0.6)	291(85.8)	48(14.2)	339
Year of study								
2014	55(55.6)	24(24.2)	14(14.1)	2(2.0)	4(4.0)	99(82.5)	21(17.5)	120
2015	48(39.7)	66(54.5)	5(4.1)	1(0.8)	1(0.8)	121(87.1)	18(12.9)	139
2016	20(28.2)	40(56.3)	8(11.3)	3(4.2)	0 (0.0)	71(88.8)	9(11.2)	80
All	123 (42.3)	130 (44.7)	27(9.3)	6 (2.1)	2 (0.7)	291(85.8)	48(14.2)	339

## Discussion

External quality control (EQC) of malaria RDT is as important as internal quality control for increasing reliability in malaria diagnosis and management of febrile patients. However, at present there are no approved EQC methods for cross-checking of used RDTs by off-site personnel because there is no guarantee that the test lines will be visible after the manufacturers’ recommended reading time. Therefore, this study reports the finding on the potential use of Fionet technology for remote monitoring of RDT test quality issues at remote clinic sites, identifying reasons contributing to laboratory errors and taking corrective actions for improvement of diagnosis, and consequently improving the management of febrile patients.

Through remote monitoring of testing at the study sites, it was observed that human errors in preparation and interpretation of RDTs results significantly affected malaria diagnosis with RDTs and hence misguided treatment of patients. A discrepancy rate of approximately 2% was observed between the results interpreted by laboratory personnel and automated analysis by the Deki Reader. The interpretation error rate reported in this study is similar to that observed from community health workers in a previous study in Kenya [24]. Among the reported discordant test results, false negative results represented over 60% implying that malaria diagnosis and treatment would be compromised and the majority of the patients would miss anti-malarial treatment [34] due to errors in diagnosis by laboratory personnel. Consequently, these patients would be in danger of developing severe disease which can be potentially fatal. Common interpretation errors (>50%) occurred because laboratory personnel could not see the presence of a single positive test line on an RDT cassette. Unfortunately, both Pf and “Pan” single positive test lines were equally highly missed during diagnosis by 65.6% and 52.9%, respectively. This finding implies that there is a high probability for patient misdiagnosis in cases where positive results are inferred for a single test line coated with antibodies for either Pf or pan antigens. It is not clearly understood why there is a high chance of laboratory personnel failing to detect a single positive test line on an RDT compared to cases where both lines were not detected properly or in cases where either of the lines were misinterpreted where both positive lines should have been reported. Therefore, further investigation is recommended on the effect of two antigens on the density of test lines because in this study it was observed that the false negativity rate was due to laboratory personnel failing to see weak positive test line (36.1%) as also previously reported [23,35]. However, another possible explanation of missing weak test lines could be presumably due to poor lighting system in the laboratory room as also previously reported [36] and confirmed by the ease of seeing positive lines when reviewing RDT images in the web portal. Therefore, investigators introduced the use of an electric table lamp to increase lighting during preparation and interpretation of RDTs; unfortunately, the impact of this intervention could not be assessed because monitoring of the quality of RDTs was done remotely.

Paradoxically, many RDT images with visible/strong positive test lines were also erroneously reported negative due to poor concentration among laboratory personnel. It has been described previously by other authors that after a period of one year post training on RDT processing and interpretation, laboratory personnel still could not safely and accurately use RDTs for malaria diagnosis [37]. In our study, our opinion is that oversimplification of RDT evaluation by laboratory personnel could also have affected RDT result interpretation.

The principal reason for reporting an RDT test result false positive was assuming the presence of a test line. Interpretation errors were commonly (>50%) attributed to assuming the presence of a single positive test line on RDT cassette while in fact it was negative. It is not clear how laboratory personnel over diagnosed these results but in our view this could potentially be due to the need for laboratory personnel to satisfy clinicians who sometimes do not believe negative test results for patients presenting with an indistinguishable fever, as also previously discussed [38].

In this study nearly 7% of the interpretation errors were due to poor RDT preparation procedures. This was higher compared to the preparation errors of 3.5% that was reported in a study done in Kenya [24]. Over 95% of the RDT images that were poorly prepared had incomplete clearance of red blood in the RDT background which hindered the interpretation process, which is similar to recently reported results [39]. In this study, few preparation errors were investigated because other common errors such as the absence of a control line, irregular control line, failure to analyze the RDT within the allowed time, use of dirty and unsupported RDTs were controlled by the use of the automated DR system which rejected the test by guiding laboratory personnel to repeat the test. This resulted in minimized errors among RDT images that were uploaded in the system and reviewed by study monitors. The proportion of processing and interpretation errors observed in the present study are also in agreement with processing error rate of 7.61% (95% CI: 6.57%, 8.76%), an interpretation error rate of 4.0% obtained from meta-analysis of over 31,000 malaria RDTs using the same Fionet technology as previously reported[40]

A significant decrease of about 97% (p<0.001) in preparation errors among discordant results was attained after three years (from 5.7% in 2014 to 0.1% in 2016) as a result of remote testing oversight by study monitors using the Fionet technology. This demonstrates that remote monitoring contributed to the improvement in RDT preparation such that very few (0.1%) errors in RDT preparation were made by laboratory personnel from all seven health facilities in the third year following remote monitoring and implementation of corrective measures. Although the preparation problems decreased noticeably, remote oversight did not work well in improving the RDT interpretation errors as demonstrated by an increased error rate of 17% in interpretation in the same period. The possible reasons behind the failure to improve RDT interpretation could include frequent replacement of trained with untrained laboratory personnel in some health facilities. We have noted that RDT testing errors increased immediately after replacements took over which does suggest a link between not having proper formal training and failure to perform testing to standard which was also documented in other studies [41]. In health facilities where laboratory personnel were replaced, study monitors trained the new staff on-site during quarterly visits and this was associated with a decline in errors.

Remote monitoring at clinic also helped to achieve timely follow-up of patients who had false negative RDT results. A follow-up rate of 49% among 669 eligible patients demonstrates the potential of the Fionet technology to improve patient follow-up through remote oversight and improvement in diagnosis. The inability to follow-up with civilian patients who had false negative results was due to long distance from the clinic coupled with a lack of communication infrastructure to notify and locate patients in remote areas in the villages of residence.

Out of all patients who were followed-up, about 86% were treated with an appropriate antimalarial therapy. The majority (92%) of febrile patients were treated within three days from their first RDT test. This achievement demonstrates also the potential of the technology to improve management of febrile patients as a result of remote oversight of laboratory personnel testing. The number of follow-up patients treated in the second year (2015) was significantly higher (p<0.001) compared to those treated in the first (2014) and third (2016). Nevertheless, follow-up of patients after detecting diagnostic errors possibly contributed to reduction in development of severe malaria among patients who missed treatment due to poor initial reporting RDT results.

## Conclusion

Fionet technology enabled remote monitoring and marked quality improvement of malaria RDT performed by laboratory personnel at 7 sites scattered across Tanzania, enhanced our ability to identify causes of RDT interpretation errors and facilitated use of effective corrective actions to improve RDT diagnosis and treatment of febrile patients in rural clinics.

The results from this study demonstrated the potential of Fionet or similar image based technology and supports its future use in external RDT quality control. It also shows potential use in remote monitoring of laboratory personnel performance preparing and interpreting RDT results while minimizing costs of conducting physical supervision to sites located in remote areas.

## Supporting information

S1 Appendix Data set for RDT external quality control of RDT using Fionet™ technology. (DTA)
